# Sound and music in video games: A systematic review of player engagement and a meta-analysis of positively valenced player experience

**DOI:** 10.1371/journal.pone.0361519

**Published:** 2026-10-27

**Authors:** Enja Heikkilä, Andrew Danso, Geoff Luck

**Affiliations:** 1 Department of Music, Art and Culture Studies, University of Jyväskylä, Jyväskylä, Finland; 2 Centre of Excellence in Music, Mind, Body and Brain, University of Jyväskylä, Jyväskylä, Finland; Damghan University, IRAN, ISLAMIC REPUBLIC OF

## Abstract

Video game sound and music have previously been found to influence player engagement and affective responses during gameplay. However, a systematic investigation into the full extent of these effects has not been conducted. This review examined the influence of music and sound on players’ affective responses and engagement during video game play, with a meta-analysis of positively valenced experience (enjoyment, fun or positive affect). Fourteen studies met the inclusion criteria, six of which provided data for the meta-analysis. Game audio was associated with more positively valenced experience than silence (*g* = 0.48, 95% CI [0.22, 0.74]; small-sample adjusted 95% CI [0.14, 0.82]). Heterogeneity (*I*² = 58%) was accounted for by one self-selected music condition; without it, the effect was smaller and homogeneous (*g* = 0.34, 95% CI [0.18, 0.50]; *I*² = 0%). In exploratory subgroup analyses, sound effects showed a consistent benefit (*g* = 0.46), whereas the added effect of music over game sound was uncertain (*g* = 0.31, adjusted 95% CI [−0.07, 0.68]). In the narrative synthesis, music and sound were often associated with higher immersion, flow and motivation. Thirteen of the 14 studies were rated at low risk of bias. Evidence that game audio improves players’ affective experience is promising but preliminary; its effect on engagement has not yet been tested quantitatively, and further research and standardisation of methods are needed.

## 1. Introduction

Player engagement in video games involves both affect and dimensions of user experience (UX; e.g., aesthetic and sensory appeal, interactivity, attention and challenge, see [1]). Research has found that music and sound can influence different domains of the player experience (PX) or game experience (GX), such as enjoyment or affective responses [2,3]. Consequently, enjoyment and affect are thought to support engagement during video game play [4] leading to improved overall PX. Despite engagement, PX and UX being prevalent areas in the study of human-computer interaction (HCI), music and sound have received comparatively little research attention. Accordingly, this review examines the current evidence on video game audio, with a meta-analysis of effects on positively valenced experience (i.e., an experience that is pleasant) and a narrative synthesis of engagement-related outcomes, distinguishing music from other game sound.

### 1.1 Theoretical background

Players’ affective responses to game audio can be conceptualized within the framework of core affect [5]: the elementary, conscious feeling of pleasure-displeasure (valence) and activation-deactivation (arousal). Core affect is present in, but not the same as, discrete emotions such as fear or joy, which are directed at an object and involve appraisal. Video game music and sound may rapidly shift players’ core affect along these dimensions during play. In addition, dual-process accounts (e.g., [6]) suggest that processing occurs through both automatic pathways (i.e., type 1 processes) and controlled, deliberate pathways (i.e., type 2 processes). This distinction points to how music may trigger rapid affective responses (e.g., heightened arousal from sound cueing or music) as well as slower, appraisal-based responses, such as enjoyment of the video game’s narrative content.

### 1.2 Player engagement in video games

Player engagement in video games is considered as an important sub-dimension of PX (e.g., due to higher in-game completion rates) along other dimensions such as enjoyment, flow, affect, immersion and presence [2]. Notably, player motivation and challenge are often considered to maintain engagement [7–9].

Video game music influences players significantly across different cognitive levels [3,10] and has been found to be significant in engaging players in-game interactions [11,12]. For instance, research has shown that music can significantly influence in-game player navigation, choices and strategy by conveying relevant information to the player [3,13,14]. The interactive nature of game sound has also been shown to be a key element in engaging players (e.g., [3]). For instance, it can be used to warn or give a hint to the player about an event. An important task that supports engagement is providing feedback to the user [15], and in video games, music and sound are often among the primary channels used to do this, either by commenting, signalling or cueing the players’ choices and actions related to their in-game activities [16,17].

### 1.3 Key constructs

We distinguish three levels of player experience. As stated, core affect is the momentary, conscious state of pleasure-displeasure (valence) and activation (arousal) [5]. Enjoyment (including “fun”) is an evaluative, positively valenced response to the game as a whole; it is appraisal-laden (i.e., depending on the player’s cognitive evaluation of the game, such as its challenge) and is typically reported after play (see, e.g., [18]). Its hedonic core corresponds to the pleasant end of the valence dimension of core affect [5]. Player engagement is the cognitive, emotional and behavioural involvement of the player in gameplay [1], expressed in states such as immersion, presence, flow and motivation [19,20].

We also distinguish music from other game sound. Music refers to the score or soundtrack, usually non-diegetic; sound effects and ambience refer to diegetic sounds of characters, objects and environments, as well as interface sounds [21]. The two may act through different pathways. For instance, music is thought to induce affect through routes such as emotional contagion, expectancy and evaluative condition (e.g., [22]), whereas sound effects carry information and feedback about game events and support in-game presence and realism [16,23].

### 1.4 Focus of the review

Given the aforementioned benefits of sound and music influencing player engagement in video games, coupled with the likelihood of music enhancing the overall player experience, a systematic investigation is warranted. A recent systematic review and meta-analysis of the effects of game design choices by Caroux and Pujol [2], showed that across 13 comparisons, only the presence of music (as opposed to its absence) had a significant effect on player enjoyment. In addition, a recent review by Bosman et al. [24] examining the effects of audio in virtual reality experiences (VR) found a lack of standardization in measuring player experiences, yet a significant context-dependent (e.g., audio increases sense of realism; hence, if high realism is a desirable outcome, the importance of audio is high) influence of audio on user experience (most specifically, pleasantness) exists. Thus, research in this area remains scant, with limited synthesis of findings, highlighting the need for a systematic review to consolidate current knowledge and clarify future directions. Accordingly, this systematic review examines the effects of music and sound on positively valenced player experience (meta-analysis) and on engagement-related states (narrative synthesis). The research questions are: (1) Does game audio, compared with its absence, make the player experience more positively valenced, and by how much? (2) Do music and sound effects differ in this effect? (3) How does game audio relate to engagement-related states such as immersion, presence, flow and motivation? (4) Which methods are most commonly used to study these effects?

## 2. Methods

This systematic review compared gameplay with music or sound against gameplay without it (or with altered audio) on players’ affective responses and engagement. This study was conducted and is reported in line with the Preferred Reporting Items for Systematic Reviews and Meta-Analyses (PRISMA) protocol [25]. The review protocol was registered on OSF before screening began (https://doi.org/10.17605/OSF.IO/TQ4EF, 31st March, 2025).

### 2.1 Eligibility criteria

We included studies that focused specifically on the effects of music and/or sound during video game play on players’ emotional engagement, including affect, arousal, or valence. Studies were eligible if they compared gameplay with music or sound to gameplay without music or sound. Studies comparing alternative audio conditions (e.g., congruent vs incongruent music) were included in the narrative synthesis; only comparisons with a no-audio condition were pooled. Only studies published in English as peer-reviewed journal articles were included. Eligible studies used experimental designs and were published from 2010 onward. All included studies were required to report technical specifications of the game context. We also included studies where music or sound was not the primary variable of interest but was nonetheless considered as part of the investigation into video game affective engagement. Studies that did not use an experimental design, conference papers, and studies not published in English or not peer-reviewed were excluded. Studies included in doctoral theses were eligible if they followed the eligibility criteria and had been published in a peer-reviewed scientific journal. Studies in Proceedings of the ACM on Human-Computer Interaction were treated as journal articles, as it is a peer-reviewed journal (e.g., one included study, see [26]).

### 2.2 Information sources

We searched the following databases: (1) PsycINFO, (2) SCOPUS, (3) Web of Science, (4) IEEE Xplore Digital Library, (5) PubMed Central, (6) ScienceDirect, (7) Cochrane Library and (8) Computing Database. Searches were conducted on 1.–2.4.2025. The database search was supplemented by forward and backward snowball searches. Specifically, the backward snowball search involved scanning the reference lists of all included articles for potential sources, while the forward snowball search identified additional studies by examining articles that cited the included studies.

### 2.3 Search strategy

A literature search was performed using terminology related to video games, music, sound, engagement, experience, immersion, and affect. Among others, the following search terms were used: (TITLE-ABS (“video” OR “virtual” OR “casual” OR “serious”)) AND (TITLE-ABS (“game*” OR game OR “gamify” OR “gamified”)) AND (TITLE-ABS (“audio” OR “sound” OR “music*”)) AND (TITLE-ABS (“emotio*” OR “engag*” OR “experience*” OR “immers*” OR “affect*” OR “valence” OR “arousal”)). Immersion was included as a search term as it is a core component of player engagement in the frameworks we adopt (see, e.g., [19,20]), as well as due to studies of game audio frequently reporting affective outcomes alongside immersion. The search strategy is described in the review registration document (https://doi.org/10.17605/OSF.IO/TQ4EF), and a glossary of search terms is provided as S1 File.

### 2.4 Selection process and data collection process

The citations of all retrieved articles were imported into Zotero, and all duplicates were manually removed. Study title and abstract were then screened by two authors (EH, AD) using ASREVIEW [27]. If the article could not be excluded based on the title or abstract, the retrieved full-text articles were then assessed for inclusion by two authors independently. Agreement at full-text stage was *k* = 0.85, and disagreements were resolved by discussion.

### 2.5 Data extraction

Effect sizes were extracted using the following rules applied to all studies. (1) The comparator was all audio present versus all audio absent; in factorial designs this was the fully on versus fully off condition, and where sound effects were present in every condition (e.g., [28,29]), music versus no music. (2) Where several positively valenced outcomes were reported, we used enjoyment, then positive affect, then fun ratings; negative affect scales were not combined with positive ones unless reverse-scored within a published composite. (3) All eligible experiments within an article were included and combined into one composite effect per article. (4) In multi-arm studies, an effect was computed for each eligible arm against the shared control, and these effects were averaged. (5) Sample sizes were the analysed *n* reported in each article; where group sizes were not reported, they were derived from the allocation procedure and reported exclusions [29]. Descriptive statistics not reported in an article were computed from the authors’ open data [30]. Data were extracted by author 1 and checked in full by author 2. Core affect, enjoyment/fun and player engagement outcomes were extracted based on the operational definitions presented in Table 1. All inputs and derivations are given in supplementary S1 and S2 Tables.

**Table 1:** Operational definitions of core affect and player engagement, outlining how each construct is conceptualised and measured.

Construct	Operational Definition	Reference
Core Affect	Core affect refers to the basic, consciously accessible feelings of pleasure–displeasure (valence) and activation–deactivation (arousal) that underpin all emotional experiences. In the context of video game music and sound, core affect is operationalized through players’ immediate self-reported emotional states (e.g., positive and negative affect, pleasantness) or physiological measures (e.g., heart rate, skin conductance).	Russell [5]
Enjoyment/fun	A positively valenced evaluative response to the gameplay experience, measured by enjoyment or fun items or subscales administered after play. Treated as a valenced outcome, distinct from engagement states.	Vorderer et al. [18]; McAuley et al. [31]
Player Engagement	Player engagement refers to the cognitive, emotional, and behavioural involvement of players during gameplay. It is considered a key sub-dimension of broader constructs such as Player Experience (PX) and User Experience (UX), and typically includes aspects such as attention, absorption, immersion, presence, and motivation. Engagement is often measured using validated scales such as the Game Engagement Questionnaire (GEQ)*.	Brockmyer et al., [19]; Jennett et al., [20]; O’Brien & Toms [1]

*Note that this is different from the Game Experiment Questionnaire (GEQ; [32]).

### 2.6 Risk of bias assessment

Risk of bias was assessed independently by two authors (AD, EH, Figs 2 and 3) using the Joanna Briggs Institute (JBI) critical appraisal checklist [33], including tools for Quasi-Experimental Appraisal (13 studies), and the Analytical Cross Sectional Studies (one study, Hutchings & McCormack, 2020) checklist. JBI items were mapped to five risk-of-bias domains following RoB 2 (S5 Table). A domain was rated low risk if all applicable items were answered yes, some concerns if any was partial or unclear, and high risk if any was no; overall judgements followed the RoB 2 algorithm. Agreement was quantified with percent agreement, Cohen’s κ and Gwet’s AC1, and differences were resolved by discussion. The JBI critical appraisal tools were chosen for their adaptability to diverse study designs, providing a structured and standardized approach to assessing risk of bias. The JBI tools were more suitable for the interdisciplinary nature of this review, encompassing experimental studies in gaming, affective and cognitive psychology.

### 2.7 Data synthesis and analysis methods

We conducted a narrative synthesis to qualitatively analyse findings across four identified categories: (1) effects of music and sound on affective responses, (2) effects of music and sound on player engagement, (3) reported methodologies, and (4) technical specifications and types of video games. Studies were categorized by their primary outcomes, and trends were identified by examining the direction and strength of effects, alongside variations in study characteristics such as participant demographics, music and sound conditions, video game genres, and methodological designs. Heterogeneity was addressed by highlighting contextual factors, including differences in technical implementation of music and sound (e.g., adaptive vs. linear sound design, built-in vs. researcher- or player-selected music) and variations in game design features (e.g., platform, game type, gameplay context).

To examine how music and sound influence affect and player engagement during video game play, we performed a meta-analysis of eligible studies. Eligible studies included those that compared video game play with music or sound to gameplay without music or without sound, using a control group and an intervention arm targeting affective responses and player engagement outcomes. Additionally, at least three studies needed to report suitable, quantifiable data to be included in a single meta-analytic cluster. For quantitative synthesis, we calculated Hedges’ *g* effect sizes and standard errors using a tool developed by Wilson [34]. Within-subjects comparisons were entered as two groups of the analysed *n*, which gives conservative standard errors [35]. Multi-arm contrasts were averaged within a study, and experiments within an article were combined by inverse-variance weighting. Effects were pooled with a DerSimonian-Laird random-effects model [36] in *R* ([37], RStudio version 2026.01.2−418 “Apple Blossom” for macOS, *metafor,* [38]), with Hartung-Knapp-Sidik-Jonkman (HKSJ) adjusted confidence intervals, recommended when studies are few [39]. Heterogeneity was assessed with *Q*, *τ*² and *I*², and a 95% prediction interval was computed. Influence was examined with leave-one-out analysis and studentised residuals [40]. Subgroup analyses by audio type, an enjoyment-only analysis and alternative extraction choices were added during revision and were not prespecified. Data and syntax files for these analyses are available via OSF: (https://doi.org/10.17605/OSF.IO/TQ4EF)

## 3. Results

### 3.1 Study selection

The initial search identified 3,486 records (Web of Science: N = 755; SCOPUS: N = 1892; PsycINFO: N = 227; Computing Database: N = 82; PubMed Central: N = 35; Cochrane Library: N = 0; ScienceDirect: N = 208; IEEE Xplore Digital Library: N = 287). Searches were conducted on 1.–2.4.2025. After removing 232 duplicate records and seven retracted records, 3,172 records were excluded for not meeting the inclusion criteria (title and/or abstract) using ASREVIEW [27]. 75 reports were sought for retrieval, and all were successfully retrieved. After full-text assessment, 61 reports were excluded for the following reasons: conference papers (*n* = 45), duplicates (*n* = 1), publication outside the specified time frame (*n* = 2), studies outside the review scope (*n* = 11), and studies not reporting relevant outcome measures (*n* = 2). No additional records were identified via snowball searches. In total, 14 studies were included in the systematic review (Fig 1). Six studies were included in the meta-analysis.

**Fig 1:**
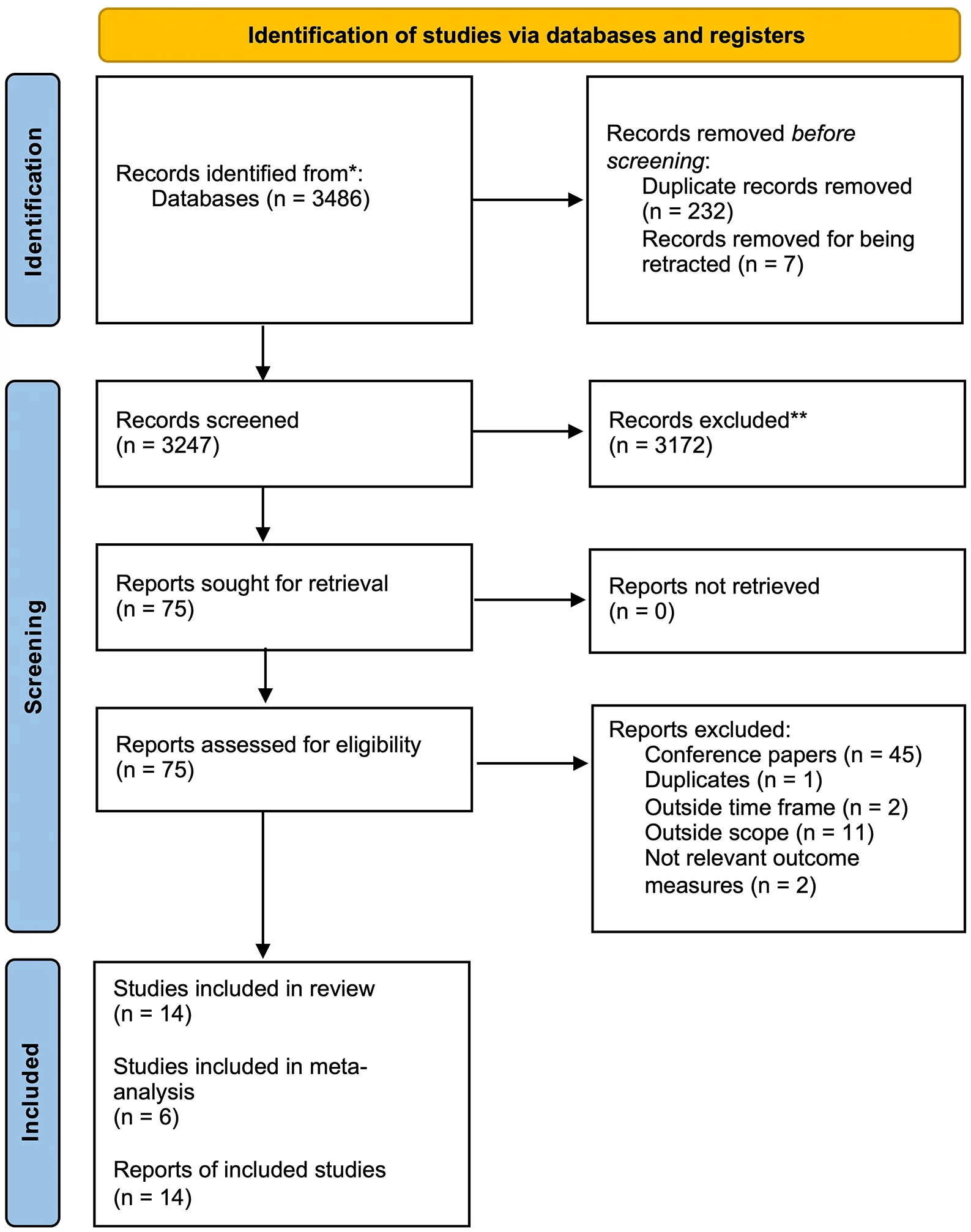
PRISMA information flow describing the screening process. *All records excluded by ASReview [27].

### 3.2 Study characteristics

The study characteristics (Table 2) include a diverse set of studies conducted in countries such as Germany (*n* = 6), Denmark (*n* = 1), the United Kingdom (*n* = 1), Australia (*n* = 1), the United States of America (*n* = 1), Japan (*n* = 1), Korea (*n* = 1), Switzerland (*n* = 1) and Canada (*n* = 1). These studies included mostly university students and adult populations. A variety of study designs are used, such as between subjects designs (*n* = 6), within subjects designs (*n* = 7) and a repeated measures design (*n* = 1). In addition, many included two or more phases or experiments (*n* = 6). Most studies were conducted using either unmodified or modified versions of commercial games (*n* = 9), whereas specifically designed games for either research (*n* = 3) or learning (*n* = 2) purposes were used in five studies. Eight studies manipulated music (six music only, two music and sound effects), and six manipulated sound effects or ambient sound only.

**Table 2:** Characteristics of the reviewed studies.

Reference	Country	N	Population	Age (y)	Study design	Audio type	Player engagement measurement	Affective response measurement	g [95% CI], positively valenced experience
Alvarez-Molina et al. [41]	DEU	27	University students	24^i^	Between subjects	Sound	Intrinsic Motivation Inventory (IMI)	IMI items (interest, enjoyment, pressure, tension)	0.18 [−0.55, 0.92]
Böttcher et al. [42]	DNK	73^d^	University students	27 ± 6.99	Within subjects	Sound	Game Experience Questionnaire (GEQ)	N/A	N/A
Cassidy & MacDonald [43]	UK^a^	70	University students	20.5 ± 2.1	Counterbalanced within subjects	Both	Post-game qualitative responses; self-designed perceived experience	Self-designed perceived experience questionnaire (enjoyment item)	1.08 [0.69, 1.47]
Graja et al. [13]	CHE	24	Adults	25^i^	Within subjects	Sound	Electrodermal activity (EDA); post-experimental questionnaire	N/A	N/A
Haehn et al. [30]	DEU	64^e^	University students	21.2 ± 3.3 (Exp 1); 22.36 ± 2.78 (Exp 2)	Counterbalanced within subjects	Sound	GEQ; player identification scale	GEQ fun (1 item in Exp 1; 5 items in Exp 2)	0.27 [−0.07, 0.62]
Hutchings & McCormack [44]	AUS	34	University students	21.4 ± 2.0	Between subjects	Music	N/A	Self-designed questionnaire	N/A
Jia & Sherrick [28]	USA	178^f^	University students	19.5 ± 1.47 (Study 1); 20.74 ± 2.61 (Study 2)	Between subjects	Music	Identification; transportation	Enjoyment scale [45,46]	0.34 [−0.02, 0.70]
Kern & Ellermeier [23]	DEU	76^g^	University students	25.5 ± 6.14 (Study 1); 22.95 ± 4.44 (Study 2)	Within subjects	Sound	Presence questionnaire	N/A	N/A
Klimmt et al. [29]	DEU	127^h^	Young men	24 ± 3.5 (Study 1); 23 ± 4 (Study 2)	Between subjects	Music	Spatial presence questionnaire	Enjoyment scale [47]; positive and negative emotions	0.38 [0.04, 0.73]
Li et al. [48]	JPN	80^j^	University students, adults	23^i^	Between subjects	Sound	Custom Game User Experience Satisfaction Scale (GUESS)	GUESS	N/A
Linek et al. [49]	DEU^b^	59	School children, adults	13.6 ± 0.89	Between subjects	Music	IMI; Flow Experience Scale	IMI items (interest, enjoyment)	N/A
Moon et al. [10]	KOR	31	University students & staff	27.3 ± 2.9	Counterbalanced within subjects	Music	GEQ	GEQ items (positive and negative affect)	N/A
Nacke et al. [50]	CAN^c^	36	University students & staff	24 ± 5	Repeated-measures factorial	Both	GEQ	GEQ items (positive and negative affect, tension)	0.53 [0.07, 1.00]
Schubhan et al. [26]	DEU	31	Adults	27.7^i^	Counterbalanced within subjects	Music	IMI; Player Experience Inventory (PXI)	IMI	N/A

Country is the first author’s affiliation. g = Hedges’ g comparing gameplay with audio against gameplay without audio (all audio present vs absent; Fig 4), pooled across arms and experiments within each article (S4 Table); N/A = not pooled or not reported. Audio type: Music = soundtrack or music; Sound = sound effects, ambient sound or audio cues; Both = music and sound effects manipulated. GEQ = Game Experience Questionnaire; IMI = Intrinsic Motivation Inventory. a Scotland, in the United Kingdom. b Study conducted in France. c Study conducted in Sweden. d Includes 17 (Study 1), 33 (Study 2), 23 (Study 3). e Includes 32 (Experiment 1), 32 (Experiment 2). f Includes 105 (Study 1), 73 (Study 2). g Includes 36 (Study 1), 40 (Study 2). h Includes 68 (Study 1), 59 (Study 2). i Approximate mean age. j Includes 40 (Phase 1), 40 (Phase 2).

### 3.3 Outcome selection and risk of bias

The original aim was to conduct meta-analyses on both core affect (valence and arousal) and player engagement outcomes. Too few studies reported comparable arousal outcomes for these to be pooled. Engagement outcomes spanned conceptually distinct constructs (immersion, spatial presence, narrative transportation, avatar identification and flow) measured with different instruments, and each construct was reported by only two or three studies (e.g., [28–30,50]).

The meta-analysis was restricted to outcomes indexing positively valenced player experience: enjoyment, fun and positive affect. All were scored so that higher values indicate a more positive experience; negative-affect items were included only where reverse-scored within a published composite (see, e.g., [30]). Six studies provided sufficient quantitative data: four measured enjoyment, one fun, and one positive affect (Table 3). As most measures were retrospective evaluations, we refer to the pooled outcomes as positively valenced experience. To check that combining these measures did not influence the result, the analysis was repeated for enjoyment measures alone.

**Table 3:** Outcome measures pooled in the meta-analysis of positively valenced experience.

Study Author	Measurement Tool	Items and scale	Construct Measured
Alvarez-Molina et al. [41]	Intrinsic Motivation Inventory, interest/enjoyment subscale [31]	Multi-item, 1–5	Enjoyment
Cassidy & MacDonald [43]	Self-designed Enjoyment Item	1 item, 0–10	Enjoyment
Haehn et al. [30]	GEQ-derived fun: Experiment 1 one positive affect item; Experiment 2 five positive and reverse-scored negative affect items [32]	1 or 5 items, 1–5	Fun
Jia & Sherrick [28]	Study 1: 7-item scale adapted from McGloin & Krcmar [45]; Study 2: 3-item scale from Oliver & Bartsch [46]	Multi-item, 1–7	Enjoyment
Klimmt et al. [29]	8-item video game enjoyment scale [47]	Multi-item, 1–5	Enjoyment
Nacke et al. [50]	GEQ positive affect subscale [32]	5 items, 0–4	Positive affect

Following the assessment of the study quality using the JBI critical appraisal checklist tools [33], the nine criteria were adapted to the five risk of bias domains found in the [51] *R* package for risk-of-bias assessments (robvis). This assessment tool evaluates the risk of bias resulting from the randomization process (D1), deviations from intended intervention (D2), missing outcome data (D3), measurement of the outcome (D4), and selection of the reported result (D5). Each domain is assessed using a judgement scale indicating high risk of bias (red cross), some concerns (yellow circle), low risk of bias (green plus), and No Information (blue question mark), with a summary of the overall risk of bias for each study presented in Fig 2. The overall risk of bias rating was determined by assigning the lowest rating observed across any of the five domains (i.e., if a high risk was present in any domain, the overall rating was also high).

**Fig 2:**
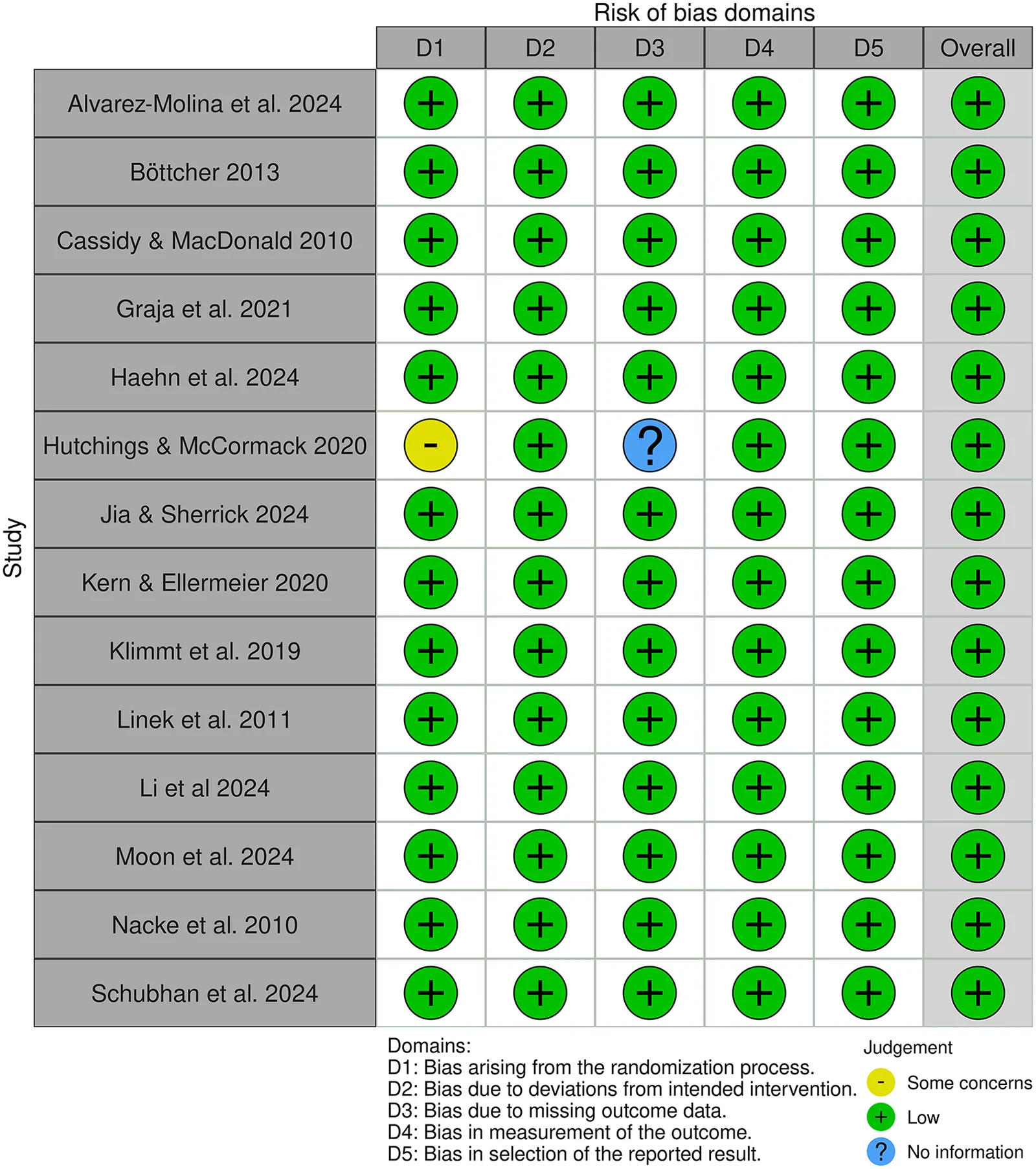
Evaluation of risk of bias in the studies included in the review, categorised across five domains from D1 to D5. An overall bias risk assessment for each study is also provided, summarising the findings across all domains [51].

Agreement between raters was 99.2% across 124 item ratings (κ = 0.97; AC1 = 0.99), with two differences, resolved by discussion. Thirteen of the 14 studies were rated low risk of bias overall and one (Hutchings & McCormack, 2020) as some concerns (Figs 2 and 3). All six studies in the meta-analysis were low risk.

**Fig 3:**
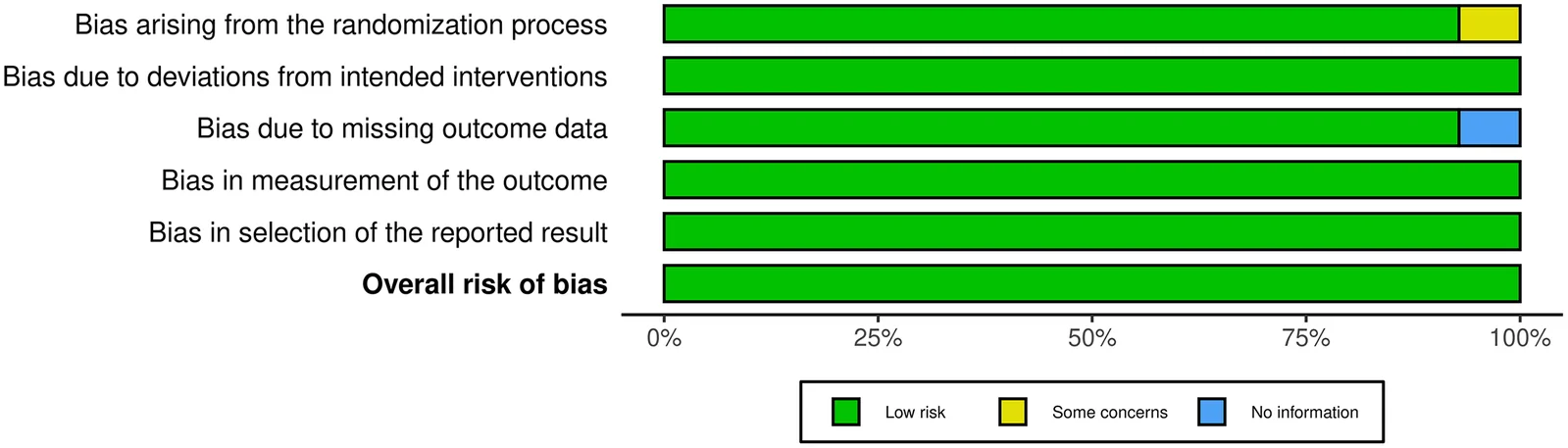
Summary of risk of bias across the 14 included studies, by domain and overall.

### 3.4 Narrative synthesis of included studies

Eight studies manipulated music (two together with sound effects) and six manipulated sound effects or ambient sound only (Table 2). Individual study findings were analysed qualitatively across four narrative categories: effects of music and sound on affective responses, effects of music and sound on player engagement, reported methodologies, as well as technical specifications and game types (see Table 4).

**Table 4:** Overview of the technical specifications and game types of the included studies, including the games used, their genres, platforms, and the sound/music conditions manipulated in each experiment.

Study	Name of game	Type of game	Platform	Conditions
Alvarez-Molina et al. [41]	*Jump ‘n’ Rhythm* [58]	2D side scrolling platformer aimed to improve rhythm skills	Computer* & headphones	(1) Auditory (2) vibrotactile
Böttcher et al. [42]	N/A	First-person perspective sword-fighting game, third person skiing game	Nintendo Wii (video game console), Macbook Pro, Wii Balance board, headphones	(1) Procedural audio (2) pre-recorded sample-based audio
Cassidy & MacDonald [43]	*Project Gotham 3* [59]	Driving game	Xbox 360 (video game console)	(1) silence (2) car sounds (3) self-selected music (4) & (5) high-arousal (6) & (7) low-arousal music
Graja et al. [13]	*P.T.* [60]	Horror game	Computer (PC), headphones	Seven scenarios with various sound qualities
Haehn et al. [30]	*League of Legends* [61]	Multiplayer online battle arena (MOBA)	Computer*, headphones	Character sounds ON/OFF, ambient sounds ON/OFF
Hutchings & McCormack [44]	*Zelda: Mystery of Solarus* [62], *Starcraft II* [63]	Action role playing game (RPG), real-time strategy game	Computer (PC)	(1) Original score (2) AMS** score
Jia & Sherrick [28]	*Life Is Strange* [64], *Highland Song* [65]	Episodic adventure game, Third-person platformer game	Computer (PC)	(1) Congruent music (2) incongruent music (3) no music
Kern & Ellermeier [23]	N/A	Virtual reality (VR) walking simulator	Oculus Rift Rk DK2 HMD (VR system) & noise-cancelling headphones	(1) No headphones (2) steps (3) steps & soundscape (4) silence (noise-cancelling) (5) soundscape
Klimmt et al. [29]	*Assassin’s Creed: Black Flag* [66], *Alien: Isolation* [67]	Adventure game & horror game	PlayStation 4 (video game console), headphones	(1) Game’s own music (2) no music
Li et al. [48]	N/A	First-person shooter (FPS)	Computer (PC), headphones	(1) Increasing volume (2) decreasing volume (3) GPR-DDA***
Linek et al. [49]	N/A	Educational adventure game regarding optics (properties of light)	Computer*	(1) Music (2) no music
Moon et al. [10]	*Sonic & All-Stars Racing Transformed Collection* [68], *Last Neighbor* [69], *Desert Golfing* [70]; *ABZU* [71]	Racing game (1), horror game (2), golf simulator (3), exploration/adventure game (4)	Computer (PC) & Genelec speakers	5 musical pieces per game session, including original score
Nacke et al. [50]	*Half-Life 2* [72]	First-person shooter (FPS)	Computer*	Music ON/OFF, sound ON/OFF
Schubhan et al. [26]	*Tetris*	Puzzle game	Computer*	No music, liked music, disliked music

* = Not further specified.

**AMS = Adaptive Music System.

***GPR-DDA = Gaussian Process Regression Dynamic Difficulty Adjustment.

#### 3.4.1 Effects of music and sound on affective responses.

Comparing the effects of soundtrack music’s presence versus absence on PX, Klimmt et al. [29] reported that music significantly increased enjoyment and positive affect in an adventure game, while Linek et al. [49] reported increased flow and motivation in a learning game. Jia & Sherrick [28] examined the effect of music’s congruence in an adventure game context, and found congruent music to elevate enjoyment, identification and transportation into the game, while incongruent music resulted in lower levels of identification and transportation. Moon et al. [10] investigated the emotional matching of musical content with the game. They describe having achieved the emotional matching by using Russell’s valence-arousal model, mapping several games and music in each emotion space through preliminary studies. They report that in a high-valence, high-arousal (HVHA) game, emotionally matching music (original soundtrack and HVHA music) was most preferred by participants. For a low-valence, low-arousal (LVLA) game, the GX was most influenced by the participant’s musical taste rather than the emotional matching between the game and music; furthermore, for the high-valence, low-arousal (HVLA) as well as low-valence, high-arousal (LVHA) games, GX’s were most optimized with emotionally matching music. Overall, the results found that the affective arousal/valence alignment between the game and the music influences GX more than the genre or familiarity of the music [10].

Studies that used music selected by the participants (e.g., [26,43]), reported increased enjoyment, liking and appropriateness linked to gameplay with preferred self-selected music. Schubhan et al. [26] also used disliked self-selected music by the participant, and found that disliked music decreased immersion. Two studies [13,50] used the definition of diegetic/non-diegetic sound to characterize the different audio qualities in their experiments. In game studies, diegetic sound refers to sound that originates from the internal game world (e.g., environment, characters and objects), whereas non-diegetic sound refers to all other sound of the game (e.g., musical score and interface sounds; [21]. Nacke et al. [50] reported that having sound on during gameplay, regardless of music, significantly affected all seven dimensions (immersion, tension, competence, flow, positive affect, negative affect and challenge) of the Game Experience Questionnaire (GEQ) [32], leading to higher ratings on positive GEQ dimensions, and lower ratings on negative GEQ dimensions. By using the term’sound’, the authors specifically refer to diegetic sound Interestingly, for tension and flow dimensions of the GEQ, the effect was dependent on the presence or absence of music [50]. Klimmt et al. [29] reported increased enjoyment and positive affect with music audible in an adventure game, while having music on in a horror game did not influence enjoyment ratings but did enhance “horror experience”, which was described by the researchers as an increase in fear or scariness, measured on a self-constructed scale derived from past research on frightening film [52]. Interestingly, Graja et al. [13] who examined a horror game experience, credited sound as the most effective factor in increasing anxiety in the horror genre, with sound facet events getting the highest relevance rates for both perceptual ratings (annotation) as well as physiological data (Galvanic Skin Response, GSR). The authors note to have mainly focused on diegetic sound (e.g., the sound of a radio) instead of non-diegetic sound [13]. In Haehn et al. [30], character sounds significantly increased immersion, avatar identification and fun, and an interaction effect between character sounds and ambient sound on fun was found. Specifically, ambient sounds increased fun only when character sounds were off [30].

#### 3.4.2 Effects of music and sound on player engagement.

Schubhan et al. [26] reported significant positive effects of music and sound on PX. Preferred self-selected music positively elevated engagement in terms of keypresses, but music had no effects on gameplay performance [26]. In Cassidy and Macdonald [43], which examined music’s effects on performance and time perception in a driving game, self-selected music improved gameplay accuracy compared to experimenter-selected music. Accuracy was measured by counting each collision with a side barrier or cone. Specifically, both self-selected and high-arousal, fast-tempo (i.e., 130 beats per minute, BPM) music resulted in the fastest lap times, yet players had the most inaccurate performance when playing under the high-arousal, fast-tempo condition, and performed most accurately with self-selected music [43]. In contrast, Schuban et al. [26] found no effect of preferred, disliked or no music on gameplay performance.

In a virtual reality (VR) walking serious game [23], having soundscape (nature sounds and ambience such as birds chirping and a bubbling stream) audible positively influenced ratings of presence, realism, involvement and distraction. In addition, using a step-reproduction algorithm which produced walking sounds simultaneously with the soundscape audio (i.e., wind, birds) audible, positively influenced participant ratings of presence and realism [23]. Li et al. [48] found that using an adaptive system, such as Gaussian Process Regression Dynamic Difficulty Adjustment (GPR-DDA) to produce audio cues, significantly improved PX in a first-person shooter (FPS) game compared to manual adjusting. Similarly, Hutchings and McCormack [44] reported higher immersion and perceived correlation of game events and music when an Adaptive Music System (AMS) was used in comparison to non-adaptive, original video game soundtrack. However, in Böttcher et al. [42], participants were not able to differentiate procedural audio from pre-recorded audio, and procedural audio had no effects on motor behaviour. Similarly, in Alvarez-Molina et al. [41], PX did not differ between vibrotactile and auditory conditions, and player performance was not affected by the presence or absence of game elements.

In two studies [28,29], congruent music positively influenced PX, and in Linek et al. [49] the presence of music compared to no music improved PX through participant ratings of enjoyment and motivation. Haehn et al. [30] reported that having character sounds audible positively influenced immersion, avatar identification and fun, but the effect was dependent on the type of gameplay at hand (e.g., free exploration versus performance-based tasks). Additionally, Nacke et al. [50] observed an interaction effect of sound effects and music on tension and flow; tension ratings were lower when both music and sound were audible and higher when only music was on, possibly indicating that having both audio types on reduced tension, whereas having only music audible did not. However, for the flow ratings, music had a positive effect on flow with sound off, yet with both sound and music on, flow ratings were lower than with only sound audible [50].

#### 3.4.3 Reported methodologies.

To measure affective responses, five studies used validated self-report questionnaires, such as the Game User Experience Satisfaction Scale (GUESS; [48]), the Positive And Negative Affect Schedule (PANAS; [29]), and the Intrinsic Motivation Inventory (IMI; [26,41,49]). Kern and Ellermeier [23] combined items from the Witmer and Singer [53] presence questionnaire and Hendrix and Barfield [54] scale. Multiple studies used GEQ [10,30,50], which is a popular questionnaire among game experience studies, however, its validity has been under debate [55,56]. Additionally, five studies [13,28,29,43,44] used self-designed scales either alone or combined with other measures. Several studies also used a perceived cognitive workload self-reported questionnaire, such as the NASA Task Load Index (NASA-TLX; [41,49]), Usability Metric for User Experience (UMUX-Lite; [41]) and player experience questionnaires, such as the Player Experience Inventory (PXI; [26)]. Physiological measures were used in three studies [10,13,50], including electroencephalography (EEG; [10]), galvanic skin response (GSR) or electrodermal activity (EDA; [13]), and a combination of electromyography (EMG) and EDA [50]. Additionally, several studies collected in-game data or keypress data [26,30,41,43,48], motion data [42] and RankTrace [57] annotation tool [13].

#### 3.4.4 Technical specifications and game types.

The included studies encompassed a diverse range of game genres, with FPS, adventure games, and platformers most commonly represented. Computers (including Personal Computers, PC) were the primary experimental platform [10,13,26,28,30,41,44,48–50], while some studies utilized consoles [29,42,43] and a VR system [23]. Auditory manipulations varied widely. Table 4 summarizes the technical specifications and characteristics of each study.

### 3.5 Meta-analysis

Six of the 14 studies provided quantitative data on positively valenced experience (enjoyment, fun, or positive affect) that could be pooled (Table 3). Engagement and arousal outcomes were not pooled and are reported in the narrative synthesis. All analyses compare gameplay with audio against gameplay without it.

#### 3.5.1 Results for positively valenced experience.

Across six studies, game audio was associated with more positively valenced experience than silence (*g* = 0.48, 95% CI [0.22, 0.74], *p* < .001; HKSJ 95% CI [0.14, 0.82], *p* = .015; Fig 4). Heterogeneity was moderate (Q = 11.93, p = .036; *τ*² = 0.06; *I*² = 58%), and the 95% prediction interval (−0.06 to 1.03) indicates that the effect in a new setting could plausibly range from null to large.

**Fig 4:**
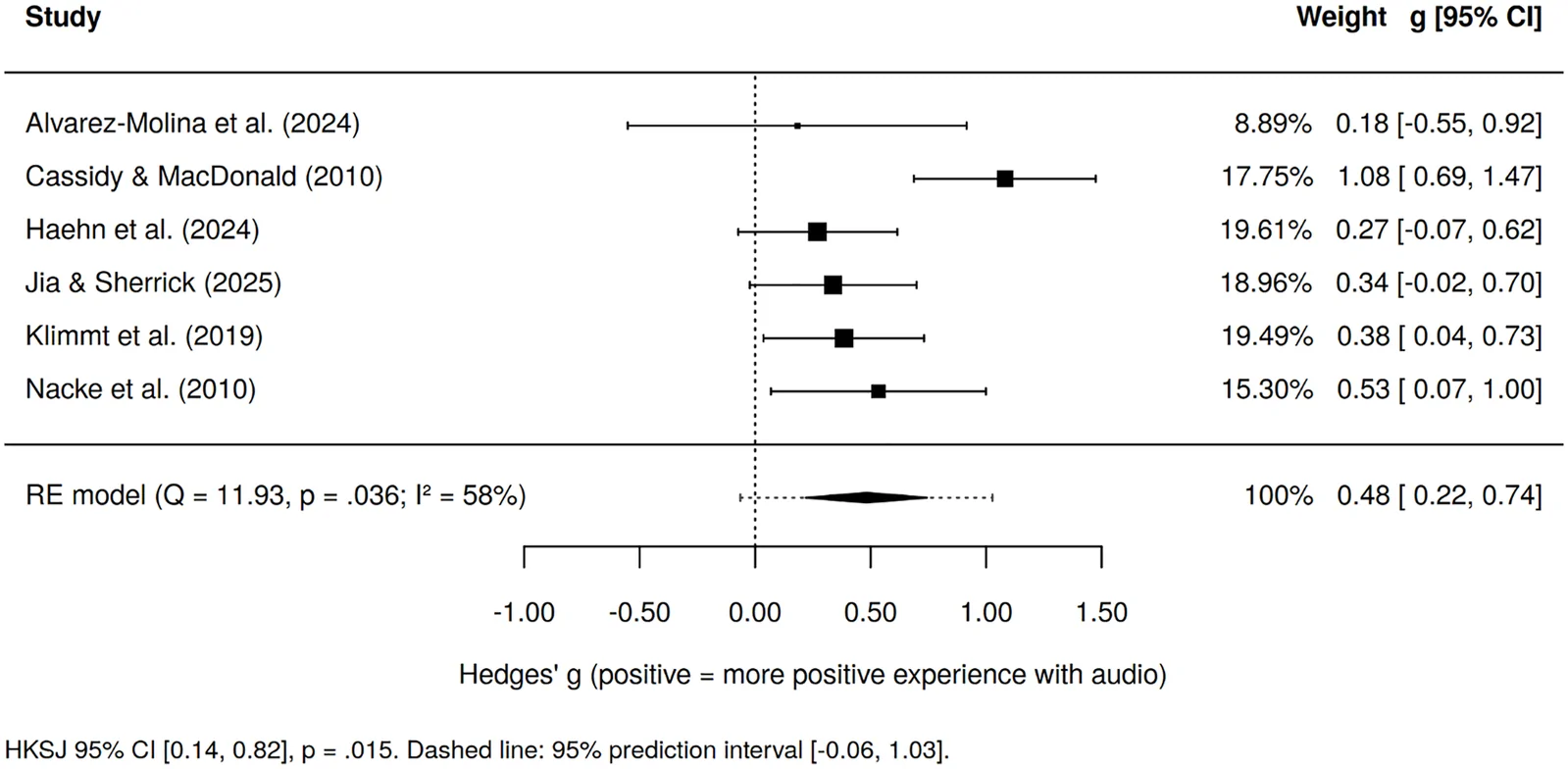
Effects of game audio on positively valenced player experience. Forest plot of Hedges’ g with 95% confidence intervals for six studies; square sizes indicate random-effects weight. Note: HKSJ = Hartung-Knapp-Sidik Jonkman adjusted confidence interval.

In leave-one-out analyses (S3 Table), the pooled estimate ranged from *g* = 0.35 to 0.53, and HKSJ-adjusted intervals excluded zero. Cassidy and MacDonald [43] was the only influential study (studentised residual = 3.30; all others below |0.8|). Its effect was driven by a single condition: enjoyment with self-selected music (M = 9.6 on a 0−10 scale) far exceeded all other conditions (2.2–3.6). Removing that condition while retaining the study gave *g* = 0.34 (95% CI [0.18, 0.50]; HKSJ 95% CI [0.24, 0.44]; *p* < .001, Fig 5) with no residual heterogeneity (Q = 1.15, *p* = .95; *I*² = 0%). The pooled estimate was similarly robust to alternative extraction choices and to within-subjects standard errors assuming *r* = .5 (*g* = 0.48–0.55 across specifications; S4 Table). Restricting the analysis to the four studies measuring enjoyment gave *g* = 0.53 (95% CI [0.13, 0.92]; *I*² = 70%). The HKSJ-adjusted interval included zero (*p* = .077); without the self-selected music condition the estimate was *g* = 0.32 (95% CI [0.13, 0.52]; *I*² = 0%).

**Fig 5:**
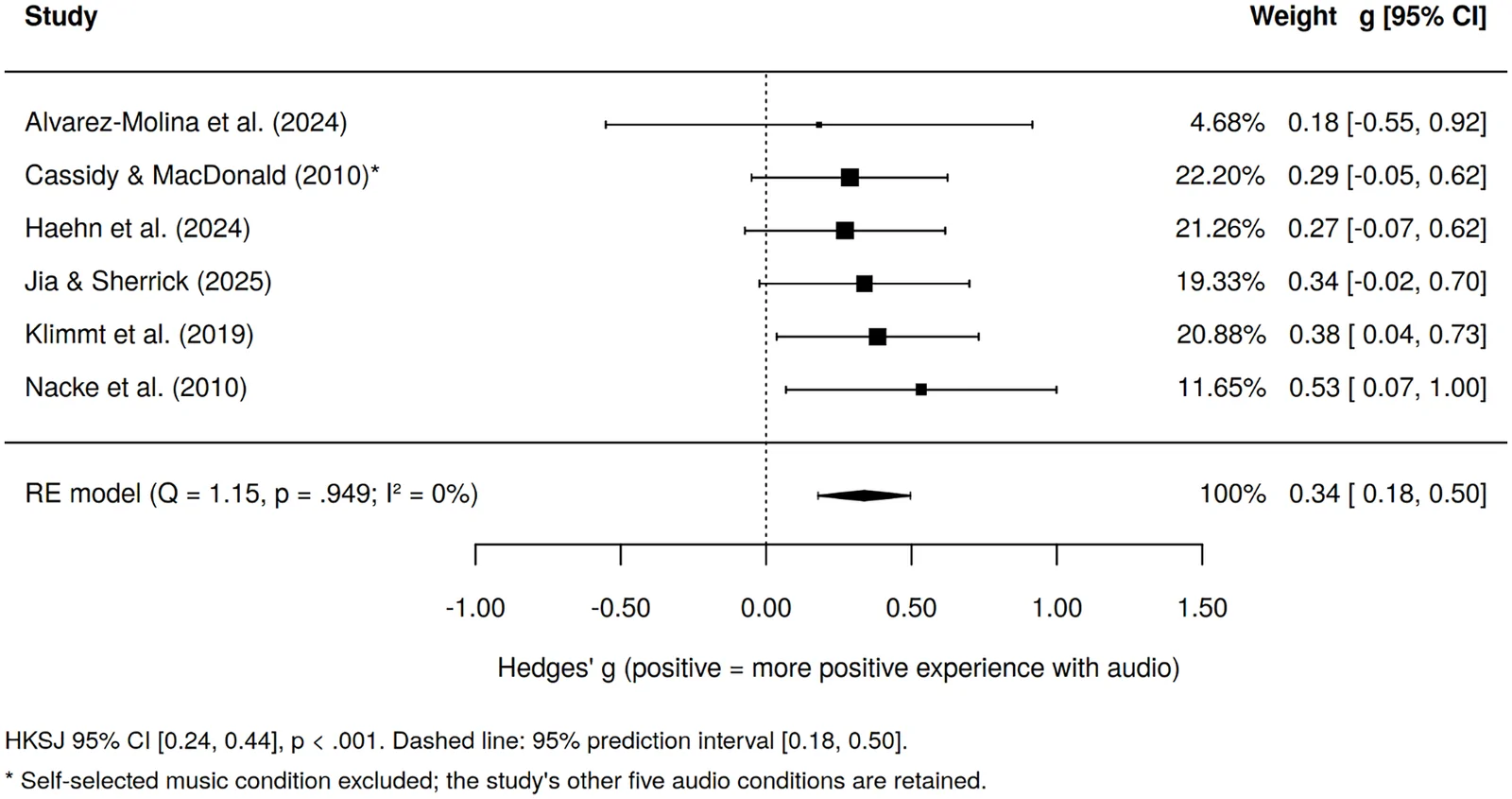
Sensitivity analysis excluding the self-selected music condition of Cassidy & MacDonald (2010). Note: The study is retained with its other five audio conditions (*).

#### 3.5.2 Sound effects and music.

Sound effects and ambient sound were associated with a more positively valenced experience than silence (*g* = 0.46, 95% CI [0.25, 0.68]; HKSJ 95% CI [0.12, 0.81], *p* = .024; *k* = 4; *I*² = 8%). Music added to game sound showed a smaller and less certain effect (*g* = 0.31, 95% CI [0.08, 0.53]; HKSJ 95% CI [−0.07, 0.68], *p* = .082; *k* = 4; *I*² = 26%; Fig 6). Within the music contrasts, effects were largest for self-selected music [43] and congruent music [28], and negative for experimenter-selected high-arousal music; excluding the self-selected condition reduced the music effect to *g* = 0.03. With four contrasts per subgroup, these estimates are exploratory.

**Fig 6:**
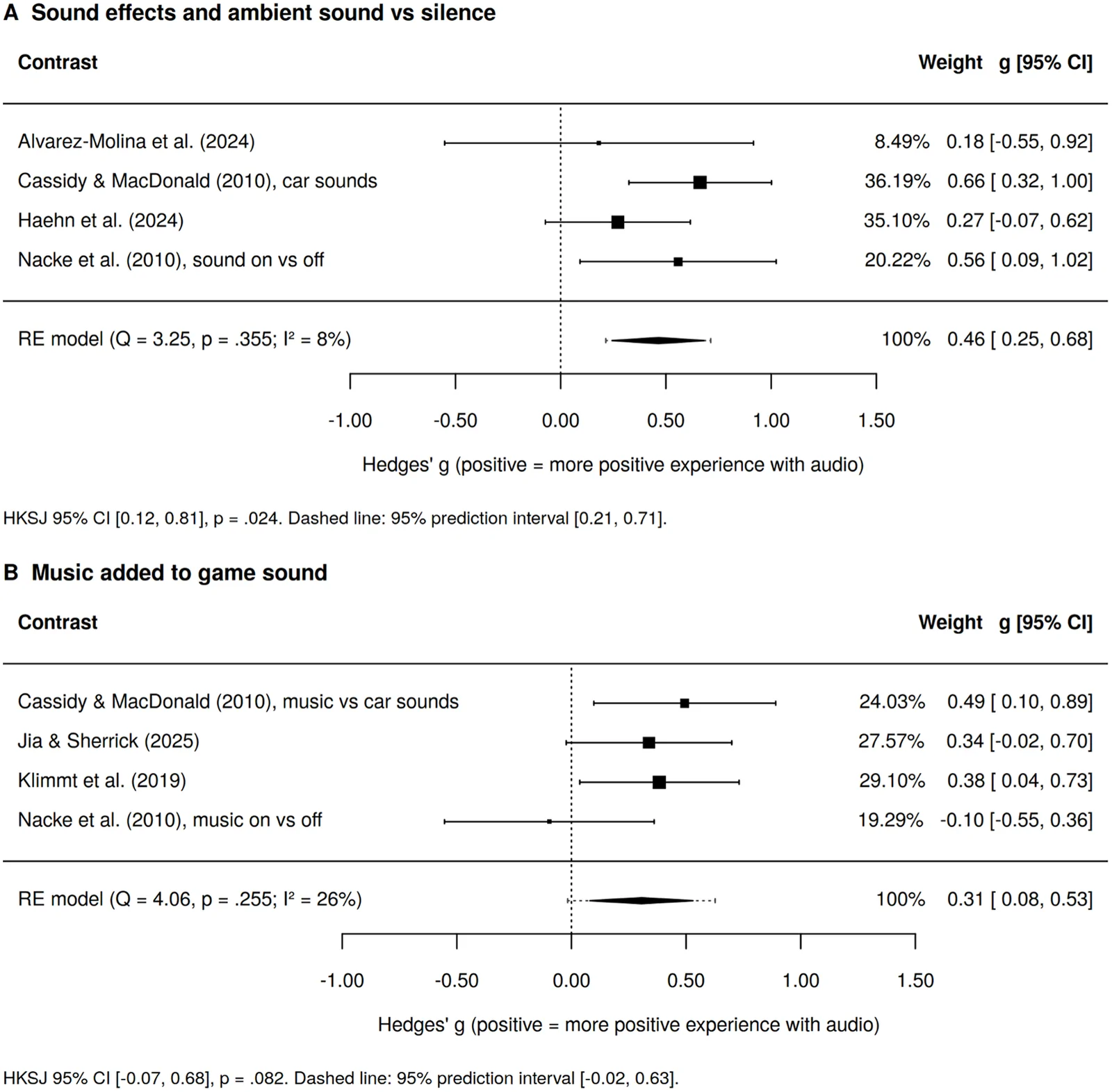
Subgroup analyses by audio type. (A) Sound effects and ambient sound versus silence. (B) Music added to game sound versus game sound alone. Cassidy & MacDonald [43] and Nacke et al. [50] contribute one contrast to each panel, the panels are not independent.

## 4. Discussion

This review systematically evaluated the influence of music and sound on players’ affective experience and engagement during video game play. Meta-analysis showed that game audio was associated with more positively valenced experience than silence, and the estimate held across alternative analytic choices, although the prediction interval included zero and the heterogeneity was accounted for by a single self-selected music condition. Exploratory subgroup analyses suggested a consistent benefit of sound effects, whereas the added benefit of music over game sound was uncertain. Narrative synthesis on player affective responses and engagement found music and sound to significantly influence PX, including enjoyment, flow, immersion, motivation and positive affect. However, three studies [10,29,30] show evidence of the effects of music and sound being dependent on game context (e.g., FPS or racing game) or experimental task (e.g., free roaming or killing enemies). The implementation of real-time adaptive audio systems – such as those responding dynamically to in-game player actions or emotional states [44,48] – resulted in significantly enhanced PX (e.g., immersion, enjoyment), provide evidence in favour for the continued development and empirical investigation of adaptive sound technology in video game environments.

Thirteen of the 14 included studies were at low risk of bias, but this concerns internal validity and does not establish certainty in the pooled estimate. The meta-analysis rests on six small laboratory studies with heterogeneous self-reported measures and English-language journal articles only, and publication bias could not be assessed formally, thus the pooled effect may be read as promising but preliminary. Although no widespread or systematic bias was evident, the incomplete reporting limits full confidence in certain findings and suggests the need for improved transparency in future research.

The effects of sound and music on positive affect and enjoyment were reported by the majority of studies and are consistent with the meta-analytic result (see, e.g., Figs 4 and 5), a small-to-moderate pooled effect. These results correspond with a positive increase in the valence dimension of Russell’s [5] core affect model, indicating that music and sound may play a key role in influencing positive emotional responses during gameplay. As player enjoyment and positive affect can elevate engagement during gameplay [4,9], it can be argued that these effects may contribute to player engagement, although this pathway was not tested quantitatively here. Enjoyment is closely linked to flow [9], which makes engagement a plausible downstream outcome.

The difference between sound effects and music, although exploratory, is informative. Sound effects provide feedback on players’ actions and information about the game world [16], and removing them made the experience less positive across all GEQ dimensions in Nacke et al. [50]. Music appeared to help mainly when it fitted the game or the player (i.e., when it was congruent with the game’s content and emotional tone, or chosen or liked by the player): effects were largest for self-selected [43] and congruent music [28], whereas disliked music reduced immersion [26] and experimenter-selected high-arousal music reduced enjoyment [43].

Notably, the findings of this review align with a recent review by Caroux and Pujol [2] who found that music presence influenced enjoyment. Furthermore, Graja et al. [13] found that sound had the strongest efficacy in inducing anxiety. According to dual-process models, music and sound in these studies appear to influence both affective and cognitive processing during gameplay (e.g., by modulating automatic Type 1, and controlled Type 2 responses). For instance, in Cassidy and MacDonald’s [43] racing game study, participant-selected (preferred) music yielded both the fastest lap times and highest accuracy, while experimenter-selected high-arousal music (130 bpm) produced similarly fast lap times but the poorest accuracy. Interestingly, participants overestimated elapsed time and inaccuracy in the preferred-music condition, whereas they underestimated elapsed time under high-arousal music [43].

One interpretation is that preferred music elevates affective valence, engaging Type 1 emotional responses (e.g., a players’ immediate affective responses) while supporting Type 2 controlled processing, thereby improving task accuracy without sacrificing response speed. In contrast, experimenter-selected high-arousal music used in Cassidy & MacDonald [43] may elevate Type 1 reflexive responses, leading to faster but less accurate performance due to reduced Type 2 monitoring and distorted time perception. Findings from Schubhan et al. [26] and Jia and Scherrick [28] indicate that affective congruence and personal relevance (e.g., self-selected music) support the complementary engagement of Type 1 (automatic, affective) and Type 2 (controlled, deliberative) processes, enhancing player performance and experience more effectively than either process alone. No included study measured these processes directly, so these interpretations are hypotheses for future work.

The majority of studies emphasize evidence of context-dependent effects, particularly in how PX and engagement are influenced by an interplay of game design choice, objectives, audio elements and player tasks. Haehn et al. [30] observed that the combination of ambient and character sounds improved PX, but only when players were allowed to freely explore the environment. The freedom to explore — as opposed to being restricted by clearly defined tasks — can enhance engagement [15]. Cumulatively, this suggests that the efficacy of audio design cannot be fully understood in isolation; it is closely tied to the player’s goals and the broader game structure. For instance, soundscapes may enhance immersion, but they are unlikely to compensate for disengaging or restrictive objectives on their own. Second, this aligns with Grimshaw et al.’s [73] assertion that sound’s impact on performance and subjective experience is modulated by multiple contextual factors — such as game features, audio presentation, player actions, and player expertise. Similarly, the importance of contextual nuance (e.g., game experience types, design choices) in game experience research has been addressed by Vahlo and Tuuri [9], who argue for models (e.g., PXI; [74]), that consider multiple functional and psychosocial aspects of gameplay – for instance, ease-of-control and mastery) accounting for contextual variability (e.g., game design choices such as gameplay mechanics). These findings are also in line with Bosman et al. [24], who emphasize the importance of contextual factors associated with player engagement.

In addition to the variety of game types, multiple questionnaires (e.g., GEQ, PANAS) as well as physiological data were used to measure player affective responses. Physiological measures were used in three studies: facial EMG and EDA [50], EEG [10], and EDA with continuous annotation [13]. Results were mixed: Graja et al. [13] found EDA responses associated with sound events, whereas Nacke et al. [50] found no physiological effects of sound or music, and in Moon et al. [10] EEG indices of arousal, valence, engagement and workload differed from rest during gameplay but barely between music conditions. This may, in part, be due to the type of physiological data gathered (Nacke et al. analysed tonic instead of phasic skin conductance; 51), or to gameplay itself driving physiological activity strongly enough to mask differences between audio conditions [10].

Regarding the effects of congruent music [28,29] and preferred music [26,43] on PX – the evidence points to potential pathways for the further study and development of affect-adaptive music systems. For example, positive findings from [26,28,29,43] suggest that player preference and affective responses could be used to determine how the music should playback and adapt throughout the gameplay experience, by using metrics such as in-game data (e.g., character movement or action data), annotation (e.g., RankTrace) or biofeedback data (e.g., heart rate), to strengthen the player’s emotional engagement and enjoyment of the game. Croissant et al.’s [75,76] recent work on advancing methodological approaches in affect-adaptive video game design revealed that an affect-adaptive game content system was able to significantly increase self-reported valence in comparison to non-adaptive audio versions of the game. This is associated with the positive results from Hutchings and McCormack [44] AMS system, who found an increase in positive affect and immersion in players, as well as the significant increase in PX when using the GPR-DDA system in Li et al. [48].

It is noteworthy that across the different studies, multiple scales such as the GEQ, PANAS as well as self-designed scales were used to assess affective responses and aspects of PX. The use of many measurement tools may distort cross-study comparisons and limit the generalizability of the evidence base, which adds to the argument for the need for greater consistency in measurement tools for affective responses and PX in video game research. Yet, recent efforts such as refined scales and models as well as systematic reviews have been made address this issue for both audio-specific and general game studies, providing potential for improved standardization related to the measurement of affective responses and PX (see, e.g., [2,24,77,78]).

## 5. Limitations and future directions

Only 6 studies were included in the meta-analysis. Despite finding that sound and music improve affective valence in our meta-analysis, the effect of arousal remains unclear. The pooled outcome combined related but distinct constructs (enjoyment, fun and positive affect), and the subgroup, enjoyment-only and sensitivity analyses were not prespecified and should be treated as exploratory. Excluding conference papers may have omitted relevant evidence, as HCI research is often published at conferences.

Based on dual process models [5,6] and the Yerkes-Dodson law [79], arousal may moderate how sound and music influences different types of cognitive and automatic engagement in gameplay (see, e.g., [43,50]). Thus, including arousal as a moderator or mediator may give future studies a richer understanding of the mechanisms through which auditory stimuli influence player experience, particularly by delineating when and how heightened physiological activation improves or impairs player engagement and performance across varying gameplay contexts. Additional limitations include the possibility of publication and retrieval bias, as only English-language studies from selected databases were included. Furthermore, although screening and data extraction were independently conducted by two reviewers, the use of automated tools and subjective judgment may have introduced bias.

In the reviewed literature, all affective valence outcomes were self-reported, introducing bias potential. Furthermore, the included studies had small convenience samples (mostly university students), which limits the generalizability of results. The wide variety of scales also makes comparison between studies more difficult. Additionally, affective responses and player experience (PX) were typically measured before or after gameplay, overlooking the dynamic and transient nature of in-game emotions, potentially experienced by the participant. Given that affective experiences can fluctuate rapidly (see, e.g., [80]), future studies should incorporate in-task measurements, such as real-time physiological monitoring (e.g., heart rate variability), continuous self-report tools (e.g., Experience Sampling Methods), or facial expression analysis, to more accurately capture moment-to-moment emotional engagement during gameplay. Affective-adaptive systems are a rising topic of research and development (see, e.g., [76,81]) and these systems could potentially tackle this issue efficiently in the future. Audio-specific research on how emotional engagement can influence player adherence and playtime may be highly worthwhile in the future. As an enjoyable game experience leads to improved engagement which then can result in longer playtime, and more game-related commercial revenue (i.e., such as item purchases) and more recruited players (see, e.g., [82]).

Several studies [26,28,44,49,50] did not disclose any information about the audio environment in their experiments, which raises concern about the transparency of reporting as well as their reproducibility. Crucially, our review shows, the sound and music conditions in audio-specific video game experiments significantly influence participants PX and affective responses within the video game. The sound and music conditions also significantly influence participant ratings of enjoyment and immersion, warranting explicit disclosure for their future reproducibility.

## 6. Conclusion

This review systematically evaluated the influence of sound and music on players’ affective responses and engagement during video game play. We found preliminary evidence that music was associated with a small-to-moderate improvement in positively valenced player experience, with a consistent benefit of sound effects and a less certain, preference-dependent benefit of music. Narratively, effects on engagement were mixed: music and sound improved immersion, presence, flow or motivation in some studies, but not in others, and effects depended on the game and the player’s task. Engagement outcomes could not be pooled. Moreover, some of the evidence suggests that adaptive audio systems, which respond to player experience, represent a promising direction for future research.

## Supporting information

S1 Table Deviations from the registered protocol. (DOCX)

S2 Table Effect-size inputs for every contrast. (DOCX)

S3 Table Leave-one-out analysis and influence diagnostics for the pooled outcome. (DOCX)

S4 Table Specification analysis: pooled estimates under alternative extraction and analysis choices. (DOCX)

S5 Table Mapping of JBI critical appraisal items to RoB 2 domains. (DOCX)

S1 File Glossary of search terms. (DOCX)

S2 File PRISMA 2020 Main Checklist. (PDF)
