# Supplementary material for: Sound and music in video games: A systematic review of player engagement and a meta-analysis of positively valenced player experience
Source: PLoS One. 2026 Oct 27;21(10):e0361519. doi: 10.1371/journal.pone.0361519 (PMC13712732; doi:10.1371/journal.pone.0361519)
Supplement: Supplementary file 2 [file pone.0361519.s001.docx]

**S1 Table**

**S1 Table. Deviations from the registered protocol.**

| Item | Registered plan | Final analysis | Reason | Stage |
| --- | --- | --- | --- | --- |
| Outcomes meta-analysed | Meta-analyses of core affect (valence and arousal) and of player engagement outcomes | One meta-analysis of positively valenced experience; arousal and engagement outcomes synthesised narratively | Too few studies reported comparable arousal outcomes; engagement constructs and instruments were heterogeneous | Original submission |
| Definition of pooled outcome | Affective valence | Positively valenced experience: enjoyment, fun or positive affect (Table 3) | Most measures were retrospective evaluations rather than momentary affect ratings | Added at revision |
| Treatment of mismatched music | Mismatched or alternative audio could serve as a proxy for a no-music control (eligibility criteria) | Used as a comparator only where a study had no silent condition. In Jia & Sherrick (2025), incongruent music was analysed as a treatment arm against no music | Incongruent music is an audio condition in its own right, and the study had a no-music control | Added at revision |
| Estimator and intervals | None prespecified | Estimator unchanged. Added HKSJ-adjusted confidence intervals and 95% prediction intervals | Conventional intervals are too narrow when the number of studies is small | Added at revision |
| Sensitivity analysis | None prespecified | Leave-one-out analysis and influence diagnostics. Exclusion of the self-selected music condition of Cassidy & MacDonald (2010), retaining the rest of the study | One influential study, driven by a single condition | Post hoc |
| Subgroup analyses by audio type | None prespecified | Exploratory subgroups: sound effects and ambient sound versus silence; music added to game sound | Music and sound effects may act through different mechanisms | Added at revision |
| Enjoyment-only analysis | None prespecified | Analysis restricted to enjoyment measures | To test whether pooling enjoyment, fun and positive affect drove the result | Added at revision |
| Effect-size extraction | Effect sizes calculated with the Campbell Collaboration calculator | Updated extraction rules applied to every study | Discrepancies in the original effect-size inputs were identified and corrected | Added at revision |
