# Supplementary material for: Sound and music in video games: A systematic review of player engagement and a meta-analysis of positively valenced player experience
Source: PLoS One. 2026 Oct 27;21(10):e0361519. doi: 10.1371/journal.pone.0361519 (PMC13712732; doi:10.1371/journal.pone.0361519)
Supplement: Supplementary file 3 [file pone.0361519.s002.docx]

**S2 Table**

**S2 Table. Effect-size inputs for every contrast.**

| Study | Contrast | Audio: M (SD) [n] | Control: M (SD) [n] | g [95% CI] |
| --- | --- | --- | --- | --- |
| Alvarez-Molina et al. (2024) | Auditory vs vibrotactile (game sound muted) | 3.40 (0.44) [14] | 3.29 (0.71) [13] | 0.18 [-0.55, 0.92] |
| Cassidy & MacDonald (2010) | Car sounds vs silence | 3.50 (1.20) [70] | 2.70 (1.20) [70] | 0.66 [0.32, 1.00] |
| Cassidy & MacDonald (2010) | Car + low-arousal music 70 bpm vs silence | 3.60 (1.20) [70] | 2.70 (1.20) [70] | 0.75 [0.41, 1.09] |
| Cassidy & MacDonald (2010) | Car + low-arousal music 130 bpm vs silence | 3.60 (1.20) [70] | 2.70 (1.20) [70] | 0.75 [0.41, 1.09] |
| Cassidy & MacDonald (2010) | Car + high-arousal music 70 bpm vs silence | 2.20 (1.00) [70] | 2.70 (1.20) [70] | -0.45 [-0.78, -0.12] |
| Cassidy & MacDonald (2010) | Car + high-arousal music 130 bpm vs silence | 2.40 (1.00) [70] | 2.70 (1.20) [70] | -0.27 [-0.60, 0.06] |
| Cassidy & MacDonald (2010) | Car + self-selected music vs silence | 9.60 (1.50) [70] | 2.70 (1.20) [70] | 5.05 [4.37, 5.73] |
| Cassidy & MacDonald (2010) | Car + low-arousal music 70 bpm vs car sounds | 3.60 (1.20) [70] | 3.50 (1.20) [70] | 0.08 [-0.25, 0.41] |
| Cassidy & MacDonald (2010) | Car + low-arousal music 130 bpm vs car sounds | 3.60 (1.20) [70] | 3.50 (1.20) [70] | 0.08 [-0.25, 0.41] |
| Cassidy & MacDonald (2010) | Car + high-arousal music 70 bpm vs car sounds | 2.20 (1.00) [70] | 3.50 (1.20) [70] | -1.17 [-1.53, -0.81] |
| Cassidy & MacDonald (2010) | Car + high-arousal music 130 bpm vs car sounds | 2.40 (1.00) [70] | 3.50 (1.20) [70] | -0.99 [-1.34, -0.64] |
| Cassidy & MacDonald (2010) | Car + self-selected music vs car sounds | 9.60 (1.50) [70] | 3.50 (1.20) [70] | 4.47 [3.85, 5.08] |
| Haehn et al. (2024) | Exp 1: all sounds on vs all off | 3.72 (1.02) [32] | 3.59 (1.07) [32] | 0.12 [-0.37, 0.60] |
| Haehn et al. (2024) | Exp 2: all sounds on vs all off | 3.90 (0.81) [32] | 3.54 (0.86) [32] | 0.43 [-0.06, 0.92] |
| Jia & Sherrick (2025) | Study 1: congruent music vs no music | 5.59 (1.19) [35] | 4.87 (1.34) [35] | 0.56 [0.09, 1.03] |
| Jia & Sherrick (2025) | Study 1: incongruent music vs no music | 4.65 (1.34) [35] | 4.87 (1.34) [35] | -0.16 [-0.63, 0.30] |
| Jia & Sherrick (2025) | Study 2: congruent music vs no music | 5.80 (1.02) [25] | 4.67 (1.58) [24] | 0.84 [0.26, 1.42] |
| Jia & Sherrick (2025) | Study 2: incongruent music vs no music | 5.04 (1.42) [24] | 4.67 (1.58) [24] | 0.24 [-0.32, 0.80] |
| Klimmt et al. (2019) | Study 1: soundtrack music vs none | 4.07 (0.69) [34] | 3.65 (0.88) [34] | 0.53 [0.05, 1.00] |
| Klimmt et al. (2019) | Study 2: soundtrack music vs none | 3.42 (0.98) [28] | 3.24 (0.56) [31] | 0.23 [-0.28, 0.73] |
| Klimmt et al. (2019) | Study 1: soundtrack music vs none | 3.52 (0.71) [34] | 3.09 (0.57) [34] | 0.66 [0.18, 1.14] |
| Klimmt et al. (2019) | Study 2: soundtrack music vs none | 3.14 (0.55) [28] | 3.07 (0.57) [31] | 0.12 [-0.38, 0.63] |
| Nacke et al. (2010) | Sound + music on vs both off | 2.06 (1.11) [36] | 1.49 (1.00) [36] | 0.53 [0.07, 1.00] |
| Nacke et al. (2010) | Music on vs off (sound on) | 2.06 (1.11) [36] | 2.16 (0.93) [36] | -0.10 [-0.55, 0.36] |
| Nacke et al. (2010) | Sound on vs off (averaged over music) | 2.11 (1.02) [36] | 1.55 (0.97) [36] | 0.56 [0.09, 1.02] |
