# Supplementary material for: Sound and music in video games: A systematic review of player engagement and a meta-analysis of positively valenced player experience
Source: PLoS One. 2026 Oct 27;21(10):e0361519. doi: 10.1371/journal.pone.0361519 (PMC13712732; doi:10.1371/journal.pone.0361519)
Supplement: Supplementary file 4 [file pone.0361519.s003.docx]

**S3 Table**

**S3 Table. Leave-one-out analysis and influence diagnostics for the pooled outcome.**

| Study omitted | g [95% CI] | HKSJ 95% CI | I^2^ (%) | Studentised residual |
| --- | --- | --- | --- | --- |
| Alvarez-Molina et al. (2024) | 0.51 [0.23, 0.80] | [0.11, 0.92] | 65 | -0.69 |
| Cassidy & MacDonald (2010) | 0.35 [0.17, 0.53] | [0.22, 0.48] | 0 | 3.30 |
| Haehn et al. (2024) | 0.53 [0.22, 0.84] | [0.10, 0.96] | 61 | -0.73 |
| Jia & Sherrick (2025) | 0.51 [0.19, 0.83] | [0.07, 0.95] | 64 | -0.46 |
| Klimmt et al. (2019) | 0.50 [0.17, 0.83] | [0.05, 0.95] | 65 | -0.31 |
| Nacke et al. (2010) | 0.47 [0.16, 0.78] | [0.03, 0.92] | 66 | 0.15 |
