# Supplementary material for: Sound and music in video games: A systematic review of player engagement and a meta-analysis of positively valenced player experience
Source: PLoS One. 2026 Oct 27;21(10):e0361519. doi: 10.1371/journal.pone.0361519 (PMC13712732; doi:10.1371/journal.pone.0361519)
Supplement: Supplementary file 5 [file pone.0361519.s004.docx]

**S4 Table**

**S4 Table. Specification analysis: pooled estimates under alternative extraction and analysis choices.**

| Specification | k | g [95% CI] | HKSJ 95% CI, p | I^2^ (%) |
| --- | --- | --- | --- | --- |
| Primary | 6 | 0.48 [0.22, 0.74] | [0.14, 0.82], .015 | 58 |
| Cassidy without self-selected music condition | 6 | 0.34 [0.18, 0.50] | [0.24, 0.44], < .001 | 0 |
| Cassidy excluded | 5 | 0.35 [0.17, 0.53] | [0.22, 0.48], .002 | 0 |
| Jia congruent music only | 6 | 0.55 [0.29, 0.81] | [0.21, 0.88], .009 | 58 |
| Klimmt positive affect instead of enjoyment | 6 | 0.49 [0.23, 0.75] | [0.15, 0.82], .014 | 58 |
| Haehn Experiment 2 only | 6 | 0.52 [0.26, 0.78] | [0.19, 0.85], .010 | 52 |
| Nacke sound main effect instead of all-on vs all-off | 6 | 0.49 [0.22, 0.75] | [0.15, 0.82], .014 | 58 |
| Within-subjects SEs with r = .5 | 6 | 0.49 [0.20, 0.78] | [0.15, 0.84], .015 | 75 |
| Enjoyment measures only | 4 | 0.53 [0.13, 0.92] | [-0.10, 1.16], .077 | 70 |
| Enjoyment only, without self-selected music condition | 4 | 0.32 [0.13, 0.52] | [0.22, 0.43], .002 | 0 |
| Sound effects subgroup | 4 | 0.46 [0.25, 0.68] | [0.12, 0.81], .024 | 8 |
| Music subgroup (music added to game sound) | 4 | 0.31 [0.08, 0.53] | [-0.07, 0.68], .082 | 26 |
