# Supplementary material for: Sound and music in video games: A systematic review of player engagement and a meta-analysis of positively valenced player experience
Source: PLoS One. 2026 Oct 27;21(10):e0361519. doi: 10.1371/journal.pone.0361519 (PMC13712732; doi:10.1371/journal.pone.0361519)
Supplement: Supplementary file 6 [file pone.0361519.s005.docx]

**S5 Table. Mapping of JBI critical appraisal items to RoB 2 domains.** A domain was rated low risk if all applicable mapped items were answered yes, some concerns if any was partial or unclear, and high risk if any was answered no; the overall judgement follows the RoB 2 algorithm (high if any domain is high, low only if all domains are low, otherwise some concerns). Items 4 (control group) and 5 (pre/post measurement) of the quasi-experimental checklist describe design features that are not required in counterbalanced within-subject or randomised post-test-only experiments and were set aside; counting them would rate all 14 studies as some concerns.

| JBI checklist | Item | Item content (paraphrased) | RoB 2 domain | Used in domain rating |
| --- | --- | --- | --- | --- |
| Quasi-experimental (9 items) | 1 | Cause and effect clearly identified (audio manipulation precedes the outcome) | D2 Deviations from intended intervention | Yes |
| Quasi-experimental (9 items) | 2 | Participants in the compared conditions were similar | D1 Randomisation process | Yes |
| Quasi-experimental (9 items) | 3 | Compared conditions treated alike apart from the audio manipulation | D2 Deviations from intended intervention | Yes |
| Quasi-experimental (9 items) | 4 | A control or comparison condition was included | D1 Randomisation process | No (set aside) |
| Quasi-experimental (9 items) | 5 | Outcome measured on more than one occasion | D4 Measurement of the outcome | No (set aside) |
| Quasi-experimental (9 items) | 6 | Follow-up complete, or attrition described and analysed | D3 Missing outcome data | Yes |
| Quasi-experimental (9 items) | 7 | Outcome measured in the same way in all compared conditions | D4 Measurement of the outcome | Yes |
| Quasi-experimental (9 items) | 8 | Outcome measured reliably (validated instrument) | D4 Measurement of the outcome | Yes |
| Quasi-experimental (9 items) | 9 | Appropriate statistical analysis | D5 Selection of the reported result | Yes |
| Analytical cross-sectional (8 items) | 1 | Inclusion criteria clearly defined | D1 Randomisation process | Yes |
| Analytical cross-sectional (8 items) | 2 | Subjects and setting described in detail | D1 Randomisation process | Yes |
| Analytical cross-sectional (8 items) | 3 | Exposure measured in a valid and reliable way | D2 Deviations from intended intervention | Yes |
| Analytical cross-sectional (8 items) | 4 | Objective, standard criteria used to measure the condition | D4 Measurement of the outcome | Yes |
| Analytical cross-sectional (8 items) | 5 | Confounding factors identified | D1 Randomisation process | Yes |
| Analytical cross-sectional (8 items) | 6 | Strategies to deal with confounding stated | D1 Randomisation process | Yes |
| Analytical cross-sectional (8 items) | 7 | Outcome measured in a valid and reliable way | D4 Measurement of the outcome | Yes |
| Analytical cross-sectional (8 items) | 8 | Appropriate statistical analysis | D5 Selection of the reported result | Yes |

**S5 Table (continued). Inter-rater agreement on risk-of-bias ratings.** Two authors rated all 14 studies independently. The two item-level differences (Graja et al., item 5; Klimmt et al., item 7, left blank by one rater) were resolved by discussion. Overall ratings after consensus: 13 low, 1 some concerns.

| Level | N | Percent agreement | Cohen’s kappa | Gwet’s AC1 |
| --- | --- | --- | --- | --- |
| Item | 124 | 99.2 | 0.97 | 0.99 |
| Domain (rule-derived) | 70 | 100 | - | - |
| Overall (rule-derived) | 14 | 100 | - | - |
