# Supplementary material for: Sound and music in video games: A systematic review of player engagement and a meta-analysis of positively valenced player experience
Source: PLoS One. 2026 Oct 27;21(10):e0361519. doi: 10.1371/journal.pone.0361519 (PMC13712732; doi:10.1371/journal.pone.0361519)
Supplement: Supplementary file 7 [file pone.0361519.s006.docx]

## S6 Supporting Information – Glossary of Search Terms

### Web of Science

TI=("audio*" OR "audito*" OR "sound*" OR "music*" OR "sonic*" OR "acoustic*" OR "ambience") AND TS=("affect*" OR "emotion*" OR "PX" OR "player experience" OR "user experience" OR "UX" OR "pleasure" OR "valence" OR "arousal" OR "engagement" OR "engag*" OR "enjoyment" OR "experienc*" OR "immersion" OR "immersive" OR "immers*" OR "presence" OR "involvement" OR "mood" OR "stress" OR "mental health" OR "perception") AND TI=("video gam*" OR "videogam*" OR "computer gam*" OR "gamif*" OR "online gam*" OR “game system*" OR "gaming system*" OR "game*" OR "nintendo" OR "xbox" OR "playstation" OR "MMPRPG" OR "interactive gam*" OR "gamer*" OR "gaming*" OR "game console*" OR "digital game*" OR "multiplayer*" OR "gameplay*" OR "casual gam*" OR "serious gam*" OR "VR gam*" OR "virtual reality" OR "VR" OR "virtual environment*" OR "player*" OR "casual videogam*" OR "interactiv*" OR "metaverse") AND PY=(2009-2026)

= 755 results

### SCOPUS

TITLE ( "audio*" OR "audito*" OR "sound*" OR "music*" OR "sonic*" OR "acoustic*" OR "ambience" ) AND TITLE-ABS ( "affect*" OR "emotion*" OR "PX" OR "player experience" OR "user experience" OR "UX" OR "pleasure" OR "valence" OR "arousal" OR "engagement" OR "engag*" OR "enjoyment" OR "experienc*" OR "immersion" OR "immersive" OR "immers*" OR "presence" OR "involvement" OR "mood" OR "stress" OR "mental health" OR "perception" ) AND TITLE ( "video gam*" OR "videogam*" OR "computer gam*" OR "gamif*" OR "online gam*" OR "game system*" OR "gaming system*" OR "game*" OR "nintendo" OR "xbox" OR "playstation" OR "MMPRPG" OR "interactive gam*" OR "gamer*" OR "gaming*" OR "game console*" OR "digital game*" OR "multiplayer*" OR "gameplay*" OR "casual gam*" OR "serious gam*" OR "VR gam*" OR "virtual reality" OR "VR" OR "virtual environment*" OR "player*" OR "casual videogam*" OR "interactiv*" OR "metaverse" ) AND PUBYEAR > 2009 AND ( LIMIT-TO ( DOCTYPE , "cp" ) OR LIMIT-TO ( DOCTYPE , "ar" ) )

= 1892 results

### PsycINFO

(TI (audio* OR audito* OR sound* OR music* OR sonic* OR acoustic* OR ambience) AND TI (video gam* OR videogam* OR computer gam* OR gamif* OR online gam* OR game system* OR gaming system* OR game* OR Nintendo OR xbox OR playstation OR MMPRPG OR interactive gam* OR gamer* OR gaming* OR game console* OR digital game* OR multiplayer* OR gameplay* OR casual gam* OR serious gam* OR VR gam* OR virtual reality OR VR OR action gam* OR adventure gam* OR virtual environment* OR player* OR casual videogam* OR interactiv* OR metaverse) AND XB (affect* OR emotion* OR PX OR player experience OR user experience OR UX OR pleasure OR valence OR arousal OR engagement OR engag* OR enjoyment OR experienc* OR immersion OR immersive OR immers* OR presence OR involvement OR mood OR stress OR mental health OR perception)) AND PY 2009-2026 AND LA english

= 227 results (with filter: peer reviewed)

### Computing Database

title("audio*" OR "audito*" OR "sound*" OR "music*" OR "sonic*" OR "acoustic*" OR "ambience") AND title("video gam*" OR "videogam*" OR "computer gam*" OR "gamif*" OR "online gam*" OR "game system*" OR "gaming system*" OR "game*" OR "nintendo" OR "xbox" OR "playstation" OR "MMPRPG" OR "interactive gam*" OR "gamer*" OR "gaming*" OR "game console*" OR "digital game*" OR "multiplayer*" OR "gameplay*" OR "casual gam*" OR "serious gam*" OR "VR gam*" OR "virtual reality" OR "VR" OR "virtual environment*" OR "player*" OR "casual videogam*" OR "interactiv*" OR "metaverse") AND abstract("affect*" OR "emotion*" OR "PX" OR "player experience" OR "user experience" OR "UX" OR "pleasure" OR "valence" OR "arousal" OR "engagement" OR "engag*" OR "enjoyment" OR "experienc*" OR "immersion" OR "immersive" OR "immers*" OR "presence" OR "involvement" OR "mood" OR "stress" OR "mental health" OR "perception") AND YR(2009-2026) AND PEER(yes)

= 82 results

### PubMed Central

((audio*[Title/Abstract] OR audito*[Title/Abstract] OR sound*[Title/Abstract] OR music*[Title/Abstract] OR sonic*[Title/Abstract] OR acoustic*[Title/Abstract] OR ambience[Title/Abstract]) AND (video gam*[Title/Abstract] OR videogam*[Title/Abstract] OR computer gam*[Title/Abstract] OR gamif*[Title/Abstract] OR online gam*[Title/Abstract] OR game system*[Title/Abstract] OR gaming system*[Title/Abstract] OR game*[Title/Abstract] OR nintendo[Title/Abstract] OR xbox[Title/Abstract] OR playstation[Title/Abstract] OR interactive gam*[Title/Abstract] OR gamer*[Title/Abstract] OR gaming*[Title/Abstract] OR game console*[Title/Abstract] OR digital game*[Title/Abstract] OR multiplayer*[Title/Abstract] OR gameplay*[Title/Abstract] OR casual gam*[Title/Abstract] OR serious gam*[Title/Abstract] OR VR gam*[Title/Abstract] OR virtual reality[Title/Abstract] OR VR[Title/Abstract] OR virtual environment*[Title/Abstract] OR player*[Title/Abstract] OR casual videogam*[Title/Abstract] OR interactiv*[Title/Abstract] OR metaverse[Title/Abstract]) AND (affect*[Title/Abstract] OR emotion*[Title/Abstract] OR PX[Title/Abstract] OR player experience[Title/Abstract] OR user experience[Title/Abstract] OR UX[Title/Abstract] OR pleasure[Title/Abstract] OR valence[Title/Abstract] OR arousal[Title/Abstract] OR engagement[Title/Abstract] OR engag*[Title/Abstract] OR enjoyment[Title/Abstract] OR experienc*[Title/Abstract] OR immersion[Title/Abstract] OR immersive[Title/Abstract] OR immers*[Title/Abstract] OR presence[Title/Abstract] OR involvement[Title/Abstract] OR mood[Title/Abstract] OR stress[Title/Abstract] OR mental health[Title/Abstract] OR perception[Title/Abstract]))

= 35 results

### Cochrane Library

audio OR audito OR sound OR music OR sonic OR acoustic OR ambience in Record Title AND affect OR emotion OR PX OR player NEXT experience OR user NEXT experience OR UX OR pleasure OR valence OR arousal OR engagement OR engag OR enjoyment OR experienc OR immersion OR immersive OR immers OR presence OR involvement OR mood OR stress OR mental NEXT health OR perception in Title Abstract Keyword AND video NEXT gam OR videogam OR computer NEXT gam OR gamif OR online NEXT gam OR game NEXT system OR gaming NEXT system OR game OR nintendo OR xbox OR playstation OR MMPRPG OR interactive NEXT gam OR gamer OR gaming OR game NEXT console OR digital NEXT game OR multiplayer OR gameplay OR casual NEXT gam OR serious NEXT gam OR VR NEXT gam OR virtual NEXT reality OR VR OR virtual NEXT environment OR player OR casual NEXT videogam OR interactiv OR metaverse in Record Title (Word variations have been searched)

= 0 results

### ScienceDirect

music + video game OR sound + video game OR audio + video game

(in Title, abstract, keywords)

= 208 results (filter added: 2009-2025)

### IEEE Xplore Digital Library

("Publication Title":music OR "Publication Title":sound OR "Publication Title":audio OR "Publication Title":sonic) AND ("Publication Title":video game* OR "Publication Title":game* OR "Publication Title":gaming* OR "Publication Title":gamer* OR "Publication Title":virtual reality)

= 287 results (filter added 2009, filter added ”conferences”)
